# Multidimensional Dysfunction in Chronic Nonspecific Low Back Pain: A Correlational Study of Key Clinical Measures

**DOI:** 10.1155/prm/4984566

**Published:** 2026-05-30

**Authors:** Zhao Wang, Fen Ju, Dexin Hu, Yuheng Lu, Chenguang Zhao

**Affiliations:** ^1^ Department of Rehabilitation Medicine, Xijing Hospital, Fourth Military Medical University, Xi’an, China, fmmu.edu.cn

**Keywords:** chronic nonspecific low back pain, correlation analysis, musculoskeletal ultrasound, surface electromyography

## Abstract

**Introduction:**

The rising incidence of chronic nonspecific low back pain (CNLBP) is placing an ever‐growing burden on healthcare systems. This study aims to investigate the multidimensional impairment characteristics of core muscle structure and function, neuromuscular control, and psychological status in patients with CNLBP and to analyze the correlations among key indicators, thereby providing a basis for individualized clinical assessment and targeted intervention.

**Methods:**

A total of 56 CNLBP patients from the outpatient department of Rehabilitation Medicine at Xijing Hospital of Fourth Military Medical University were enrolled between June 2024 and January 2025. Additionally, 30 healthy volunteers were recruited as controls. Demographic data were collected. Assessments were conducted across four dimensions: pain and function, muscle morphology, neuromuscular control, and sleep and psychological status. Differences in indicators between groups were compared, and correlation analyses among indicators were performed within the CNLBP group.

**Results:**

In the CNLBP group, the pressure pain threshold (*p* = 0.012), maximum voluntary extension of back extensors (*p* < 0.001), cross‐sectional area (CSA) of the multifidus muscle (*p* < 0.001), thickness change rate of the multifidus muscle (*p* < 0.001), and flexion–relaxation ratio (FRR) (*p* < 0.001) were significantly lower than those in the healthy control group. In contrast, the finger‐to‐floor distance (*p* = 0.040), Pittsburgh Sleep Quality Index score (*p* = 0.004), Generalized Anxiety Disorder‐7 score (*p* = 0.005), and Patient Health Questionnaire‐9 score (*p* = 0.021) were significantly higher. Correlation analysis within the CNLBP group revealed that the FRR was negatively correlated with the NRS score (*r* = −0.283, *p* = 0.038) and the Oswestry Disability Index (ODI) score (*r* = −0.345, *p* = 0.011). The thickness change rate of the multifidus muscle was negatively correlated with the ODI score (*r* = −0.285, *p* = 0.037), but its correlation with the Numeric Rating Scale (NRS) score was not significant (*r* = −0.172, *p* = 0.214). The CSA of the multifidus muscle showed no significant correlation with the FRR (*r* = 0.061, *p* = 0.655). In contrast, a significant positive correlation was identified between the FRR and the rate of thickness change in the multifidus (*r* = 0.301, *p* = 0.024).

**Conclusion:**

This study confirms that CNLBP involves multidimensional impairments, including pain perception and function, multifidus muscle morphology and activation function, neuromuscular regulation of the erector spinae, and sleep quality and psychological status. The identified correlations between FRR and both NRS and ODI scores provide a crucial basis for formulating individualized rehabilitation plans and for subsequent longitudinal research.

**Trial Registration:** Chinese Registry of Clinical Trials: ChiCTR2400093968

## 1. Introduction

Epidemiological studies indicate that low back pain (LBP) is a leading cause of disability worldwide. In 2020, approximately 619 million people globally were affected by LBP, and this number is projected to reach 843 million by 2050. Notably, nonspecific low back pain (NSLBP) accounts for approximately 90%–95% of all LBP cases [1, 2]. The etiology of NSLBP is complex, with most cases lacking definitive imaging abnormalities, posing significant challenges for clinical diagnosis and management. Therefore, a multidimensional investigation to untangle its underlying causes has become a critical issue in the medical field.

Current research widely posits that structural and functional degeneration of the lumbar stabilizing muscles is a significant contributing factor to chronic nonspecific low back pain (CNLBP) [3]. As a core stabilizer, morphological alterations of the multifidus muscle have garnered considerable attention. Studies have shown that patients with LBP frequently exhibit signs of atrophy in the multifidus, including a reduced cross‐sectional area (CSA) and fatty infiltration [4]. Concurrently, decreased strength of the back extensor muscles and restricted trunk range of motion are common manifestations of functional impairment [5]. Regarding neuromuscular control, the absence or delay of the flexion–relaxation phenomenon is a typical characteristic of CNLBP, reflecting a disturbance in the dynamic stability control of the spine [6]. However, the intrinsic relationships among these indicators remain unclear.

Given that existing research is predominantly limited to the analysis of single parameters and lacks a systematic integration of “symptoms–structure–function,” this study aims to employ musculoskeletal ultrasound imaging and surface electromyography (sEMG) to compare differences in multifidus muscle morphology, activation capacity, and neuromuscular control between patients with CNLBP and healthy individuals. The objective is to provide a theoretical basis for the precise prevention, control, and development of rehabilitation strategies for CNLBP.

## 2. Materials and Methods

### 2.1. Study Subjects

The study subjects consisted of patients with CNLBP who attended the outpatient clinic of the Department of Rehabilitation Medicine at Xijing Hospital of Fourth Military Medical University and were enrolled between June 2024 and January 2025. Age‐ and sex‐matched healthy volunteers were concurrently recruited as healthy control (HC). This study was approved by the Ethics Committee of Xijing Hospital, the Fourth Military Medical University (Approval No.: KY20242185‐F‐1) and registered with the Chinese Clinical Trial Registry.

### 2.2. Inclusion and Exclusion Criteria for CNLBP Patients

Inclusion criteria: (1) age 20–60 years; (2) meeting the diagnostic criteria for NSLBP [7]; (3) symptom duration ≥ 12 weeks; (4) Numeric Rating Scale (NRS) score ≥ 3; (5) ability to cooperate with treatment and assessments; and (6) provision of written informed consent.

Exclusion criteria: (1) LBP caused by specific spinal pathologies (spinal stenosis, spondylolisthesis, vertebral body fracture, osteoporosis, tumor, tuberculosis, etc.); (2) history of spinal surgery; (3) LBP related to visceral diseases: urological disorders (kidney stones, urinary tract stones, pyelonephritis, etc.), gynecological diseases (endometrioma, etc.), and others (intra‐abdominal lesions, retroperitoneal lesions, etc.); (4) circulatory system diseases: abdominal aortic aneurysm, dissecting aneurysm, severe cardiovascular and cerebrovascular diseases, hypertension, etc.; (5) psychiatric disorders; (6) cognitive impairments, illiteracy, or communication barriers; (7) current use of analgesic or muscle relaxant medications; and (8) local skin lesions or paresthesia at the measurement sites.

### 2.3. Basic Information

General patient information was recorded, including demographic characteristics such as age, sex, marital status, lifestyle habits, and body mass index (BMI).

### 2.4. Pain and Function Evaluation

(1) NRS: Patients described their pain intensity using an 11‐point NRS based on their pain perception [8]. (2) Pressure pain threshold (PPT): With participants in a prone position, PPT was measured using a Baseline digital pressure algometer. Pressure was applied orthogonally at 1 kg/s to two points 5 cm bilaterally from the lumbar midline on the intercristal (Jacoby) line until pain was reported. The mean of three trials, separated by 30‐s intervals, was taken as the final value [9]. (3) Maximal isometric back extensor strength: It was measured using a MicroFET3 portable digital muscle tester. Following a brief warm‐up, participants sat with hips and knees flexed 90°, arms crossed. The force pad was centered at the midpoint between the superior scapular angles. Participants performed maximal back extension for 3–5 s; the test was repeated three times with 1‐min intervals, and the maximum value was recorded [10]. (4) Finger‐to‐floor distance (FFD): Participants stood barefoot on a flat surface, slowly bent forward as far as possible with arms hanging naturally and fingers reaching toward the floor, while keeping the knees straight. The vertical distance from the longest fingertip to the floor was measured. (5) Oswestry Disability Index (ODI): This index includes 10 aspects: pain intensity, personal care, lifting, walking, sitting, standing, sleeping, sex life, social activities, and traveling. Each item is scored from 0 to 5, with a total score ranging from 0 (*no disability*) to 50 (*complete disability*). A higher score indicates greater functional disability [11].

### 2.5. Muscle Morphological Assessment

Multifidus muscle thickness and CSA were measured bilaterally using a Mindray MT3 ultrasound device. With participants prone, the L4 spinous process was identified. Thickness was measured longitudinally from the L4/L5 facet joint apex to the thoracolumbar fascia during relaxation and during a resisted trunk lift holding a 1‐kg dumbbell. The thickness change rate was calculated as (contracted − relaxed)/relaxed [12, 13]. CSA was measured transversely at the L4 level in the relaxed state. The mean of three trials per side was used, and bilateral averages were analyzed [14].

### 2.6. Neuroelectrophysiology Examination

sEMG was recorded from bilateral lumbar erector spinae using a BTS FreeEMG300 system. Electrodes were placed 5 cm lateral to the L3–L4 spinous processes following skin preparation (shaving, abrasion, and cleaning with 75% alcohol). Participants performed a trunk flexion–extension task paced by a metronome, comprising four phases: upright relaxation, flexion, full‐flexion hold, and extension. Three trials were completed with 3‐min rests between trials. The average EMG amplitude (AEMG) during flexion and full‐flexion phases was used to calculate the flexion–relaxation ratio (FRR) as follows: FRR = AEMG_flexion_/AEMG_full-flexion_. Given the bilateral synergy during movement, left‐ and right‐side FRR values were averaged to produce a single FRR per participant for statistical analysis [15].

### 2.7. Sleep and Psychology Assessment

(1) Pittsburgh Sleep Quality Index (PSQI): A 0–21‐point scale assessing overall sleep quality, with higher scores indicating worse sleep [16]. (2) Generalized Anxiety Disorder‐7 (GAD‐7): Anxiety was screened using this 7‐item scale, scored 0–21, where higher scores correspond to more severe anxiety [17]. (3) Patient Health Questionnaire‐9 (PHQ‐9): Depressive symptoms over the past two weeks were self‐rated; total scores range from 0 to 27, with higher scores reflecting greater severity [17].

### 2.8. Statistical Analysis

Data analysis was performed using SPSS Version 27.0. The normality of continuous data was assessed using the Shapiro–Wilk test. Normally distributed data are presented as mean ± standard deviation (mean ± SD), and comparisons between groups were performed using the independent *t*‐test. Non‐normally distributed data are presented as median (Q1, Q3), and comparisons between groups were performed using the Mann–Whitney *U* test. Ordinal and categorical data are presented as *n* (%), and comparisons between groups were performed using the Mann–Whitney *U* test, *χ* ^2^ test, or Fisher’s exact test as appropriate. To assess the robustness of the results, a sensitivity analysis was conducted using propensity score matching (PSM) to perform a 1:1 nearest‐neighbor match between the CNLBP group and the HC group, with a caliper value set at 0.2. The matching variables included age, gender, marital status, smoking status, drinking status, and BMI. The balance after matching was evaluated using standardized mean differences (SMDs), with a value less than 0.1 considered acceptable. Correlation analysis was conducted using Pearson or Spearman correlation based on data distribution. The criterion for statistical significance was set at *p* < 0.05.

## 3. Results

A total of 86 participants were enrolled in the study, including 56 (65.1%) in the CNLBP group and 30 (34.9%) in the HC group.

### 3.1. Baseline Characteristics

A comparison of baseline characteristics between the two groups revealed no statistically significant differences in age, gender, marital status, or BMI (*p* > 0.05). A statistically significant difference was observed in smoking habits between the CNLBP group and the HC group (*p* = 0.003). No significant difference was found in the distribution of drinking status between the two groups (*p* = 0.362). In summary, the demographic and basic characteristics of the two groups were comparable, except for a higher proportion of smokers in the CNLBP group. Details are presented in Table 1.

**TABLE 1:** Demographic characteristics of the HC and CNLBP groups.

Characteristic	HC group *N* = 30 (34.9%)	CNLBP group *N* = 56 (65.1%)	*p* value
Age (year), mean ± SD	33.7 ± 8.6	34.7 ± 6.8	0.741^a^
Gender, *n* (%)			0.064^b^
Male	25 (83.33)	36 (64.29)	
Female	5 (16.67)	20 (35.71)	
Marital status, *n* (%)			0.812^b^
Married	19 (63.3)	34 (60.7)	
Living alone	11 (36.7)	22 (39.3)	
Smoking status, *n* (%)			0.003^c^
Never	19 (63.33)	15 (26.79)	
Former	8 (26.67)	17 (30.36)	
Now	3 (10.00)	24 (42.86)	
Drinking status, *n* (%)			0.362^c^
Never	7 (23.33)	10 (17.86)	
Former	20 (66.67)	33 (58.93)	
Current	3 (10.00)	13 (23.21)	
BMI (kg/m^2^), mean ± SD	22.81 ± 2.17	23.34 ± 2.02	0.261^a^

*Note:* The statistical tests employed included the following: ^a^independent samples *t*‐test; ^b^chi‐square test; ^c^Fisher’s exact test.

Abbreviations: BMI, body mass index; CNLBP, chronic nonspecific low back p HC, healthy control.

### 3.2. Comparative Analysis Between the HC and CNLBP Groups

As shown in Table 2, compared with the HC group, the CNLBP group exhibited significant differences in dimensions, including pain perception, trunk muscle strength and range of motion, morphological structure and activation capacity of the multifidus muscle, modulation of erector spinae contraction and relaxation, and sleep quality and psychological/emotional state. Regarding pain perception, the PPT was significantly lower in the CNLBP group than in the HC group (*p* = 0.012) (Figure 1(a)), indicating enhanced mechanical pain sensitivity in patients. In terms of trunk functional performance, the CNLBP group demonstrated significant functional impairment: the maximum isometric back extensor strength was significantly reduced (*p* < 0.001) (Figure 1(b)), and the FFD was significantly increased (*p* = 0.040) (Figure 1(c)), suggesting weakened back muscle strength and restricted trunk flexion mobility.

**TABLE 2:** Comparison of related variables between the HC and CNLBP groups.

Items	HC group *N* = 30 (34.9%)	CNLBP group *N* = 56 (65.1%)	*p* value
PPT (kg), mean ± SD	2.47 ± 0.93	1.94 ± 0.87	0.012^a^
MIBES (N), mean ± SD	204.93 ± 21.24	181.12 ± 14.97	< 0.001^a^
FFD (cm), mean ± SD	13.13 ± 4.19	15.68 ± 5.76	0.040^a^
MF‐CSA (cm^2^), mean ± SD	5.67 ± 1.29	4.30 ± 0.96	< 0.001^a^
MF thickness change rate (%), mean ± SD	42.00 ± 7.00	32.00 ± 7.00	< 0.001^a^
FRR, median (IQR)	4.65 (3.81, 5.15)	1.54 (0.93, 2.22)	< 0.001^b^
PSQI, mean ± SD	2.63 ± 1.66	4.07 ± 2.34	0.004^a^
GAD‐7, mean ± SD	0.64 ± 0.36	1.47 ± 0.43	0.005^a^
PHQ‐9, median (IQR)	1.00 (0.07, 2.84)	3.03 (1.03, 5.07)	0.021^b^

*Note:* The statistical tests employed included the following: ^a^independent samples *t*‐test; ^b^Mann–Whitney *U* test.

Abbreviations: CNLBP, chronic nonspecific low back pain; FFD, finger‐to‐floor distance; FRR, flexion–relaxation ratio; GAD‐7, Generalized Anxiety Disorder‐7; HC, healthy control; MF‐CSA, the cross‐sectional area of the multifidus; MIBES, maximal isometric back extensor strength; PHQ‐9, Patient Health Questionnaire‐9; PPT, pressure pain threshold; PSQI, Pittsburgh Sleep Quality Index.

FIGURE 1Comparison of PPT, MIBES, and FFD between the HC group and the CNLBP group. (a) Comparison of PPT between the two groups; (b) comparison of MIBES between the two groups; (c) comparison of FFD between the two groups. HC: healthy control; CNLBP: chronic nonspecific low back pain; PPT: pressure pain threshold; MIBES: maximal isometric back extensor strength; FFD: finger‐to‐floor distance. ^∗^ *p* < 0.05, ^∗∗∗^ *p* < 0.001.

**Figure:**
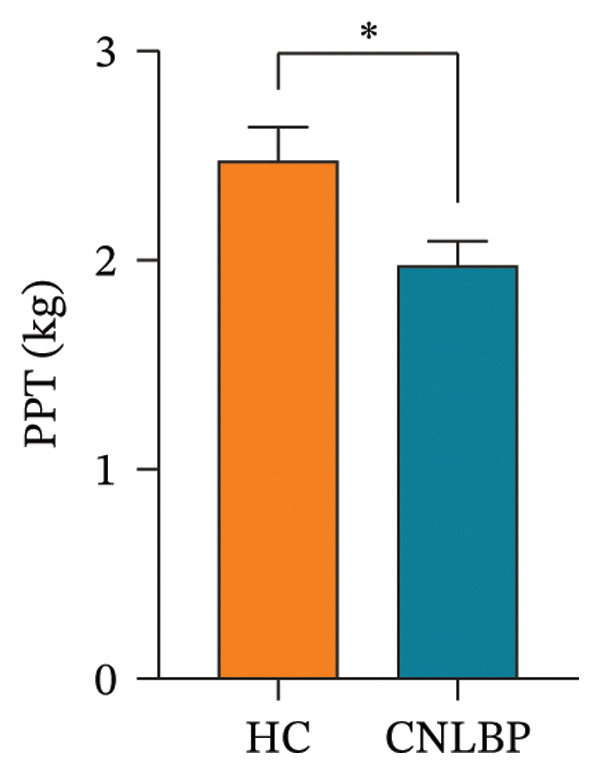
(a)

**Figure:**
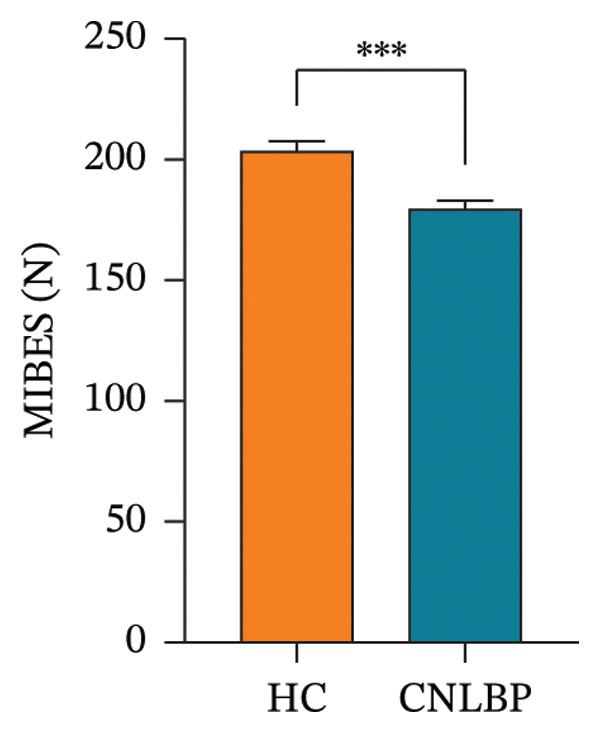
(b)

**Figure:**
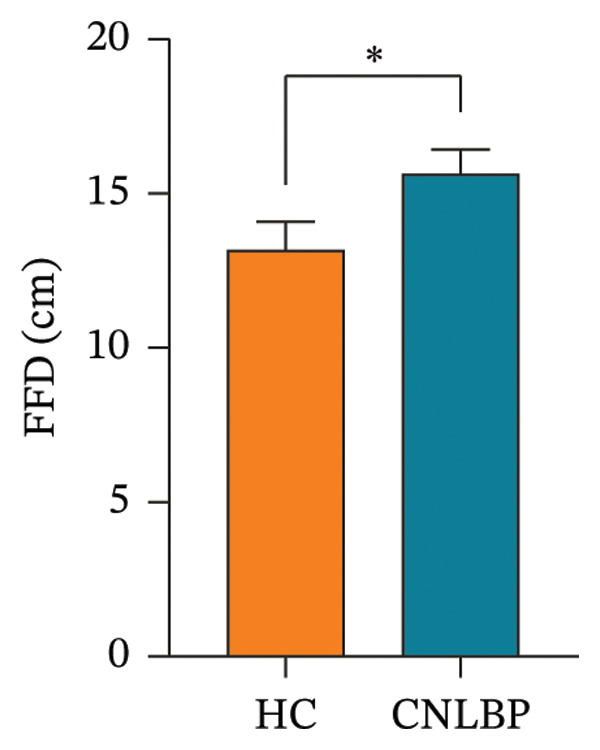
(c)

**FIGURE 2:**
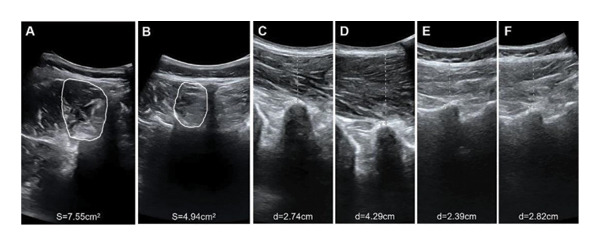
Representative ultrasonographic images of the multifidus muscle from one subject in the HC group and one subject in the CNLBP group. (A) CSA of the multifidus in the HC group; (B) CSA of the multifidus muscle in the CNLBP group; (C) thickness of the multifidus at rest in the HC group; (D) thickness of the multifidus during contraction in the HC group; (E) thickness of the multifidus at rest in the CNLBP group; (F) thickness of the multifidus during contraction in the CNLBP group; HC: healthy control; CNLBP: chronic nonspecific low back pain; CSA: cross‐sectional area.

Regarding muscle structural characteristics, the CNLBP group exhibited structural alterations and functional decline in the multifidus muscle: The CSA of the multifidus was significantly reduced (*p* < 0.001) (Figures 2(A), 2(B), and 3(a)), and the rate of thickness change during contraction was significantly decreased (*p* < 0.001) (Figures 2(C), 2(D), 2(E), and 2(F), 3(b)), indicating muscle atrophy and impaired contractile function. In sEMG parameters, the FRR was significantly lower in the CNLBP group (*p* < 0.001) (Figure 3(c)), reflecting a loss of the normal muscle relaxation pattern in the lumbar region during trunk flexion.

FIGURE 3Comparison of the MF‐CSA, multifidus thickness change rate, and FRR between the HC group and the CNLBP group. (a) Comparison of the MF‐CSA between the two groups; (b) comparison of multifidus thickness change rate between the two groups; (c) comparison of FRR between the two groups. HC: healthy control; CNLBP: chronic nonspecific low back pain; MF‐CSA: the cross‐sectional area of the multifidus; FRR: flexion–relaxation ratio. ^∗∗∗^ *p* < 0.001.

**Figure:**
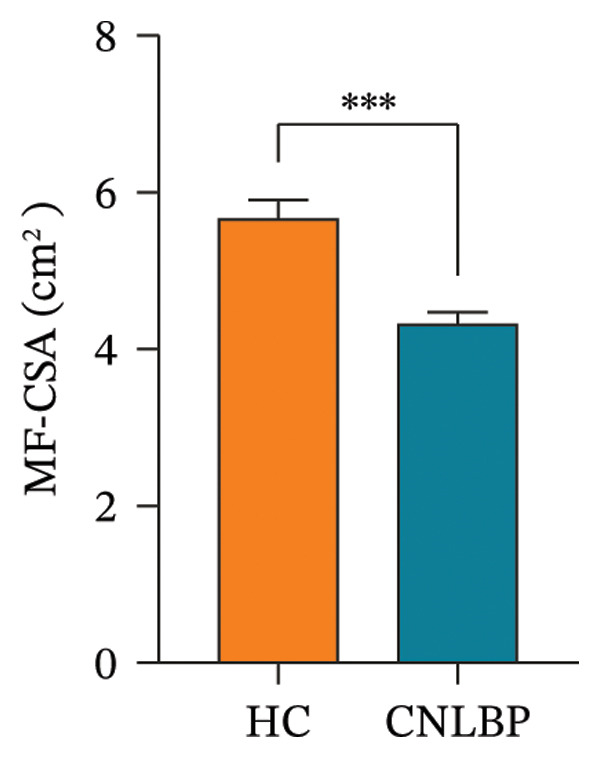
(a)

**Figure:**
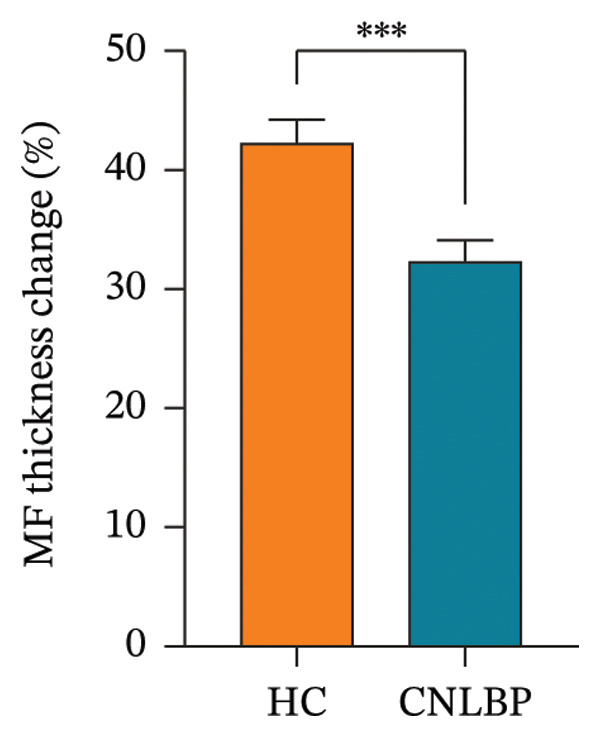
(b)

**Figure:**
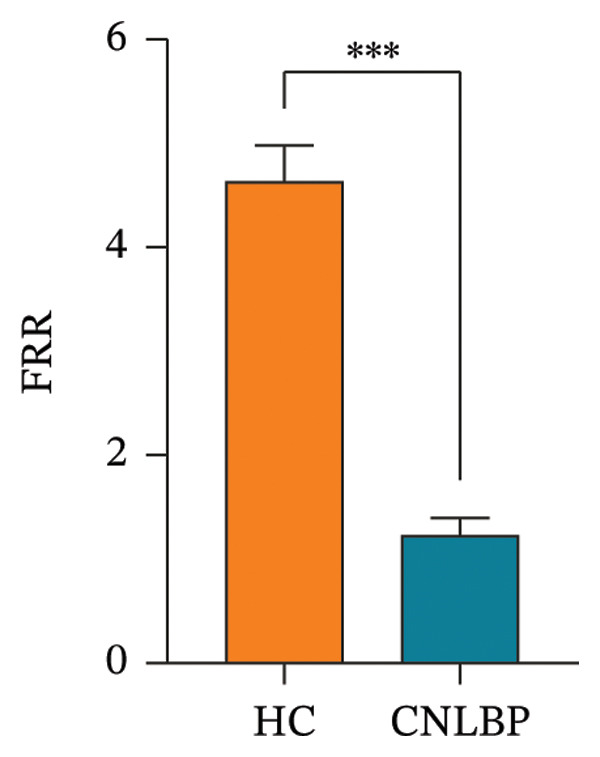
(c)

Furthermore, in sleep and psychological assessments, the CNLBP group scored significantly worse than the HC group on the PSQI (*p* = 0.004) (Figure 4(a)), the GAD‐7 (*p* = 0.005) (Figure 4(b)), and the PHQ‐9 (*p* = 0.021) (Figure 4(c)), indicating that patients with CNLBP experience significant sleep disturbances, anxiety symptoms, and depressive mood.

FIGURE 4Comparison of PSQI, GAD‐7, and PHQ‐9 scores between the HC group and the CNLBP group. (a) Comparison of PSQI scores between the two groups; (b) comparison of GAD‐7 scale scores between the two groups; (c) comparison of PHQ‐9 scores between the two groups. HC: healthy control; CNLBP: chronic nonspecific low back pain; PSQI: Pittsburgh Sleep Quality Index; GAD‐7: Generalized Anxiety Disorder‐7; PHQ‐9: Patient Health Questionnaire‐9. ^∗^ *p* < 0.05, ^∗∗^ *p* < 0.01.

**Figure:**
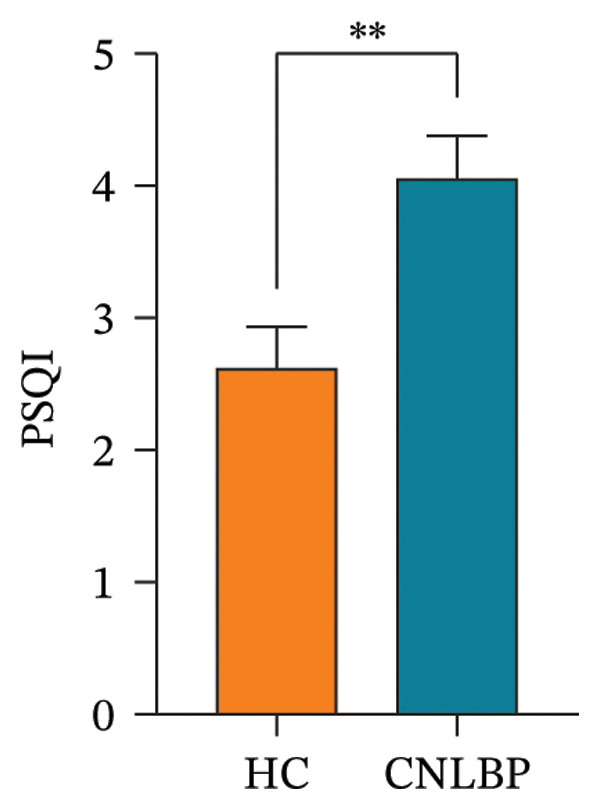
(a)

**Figure:**
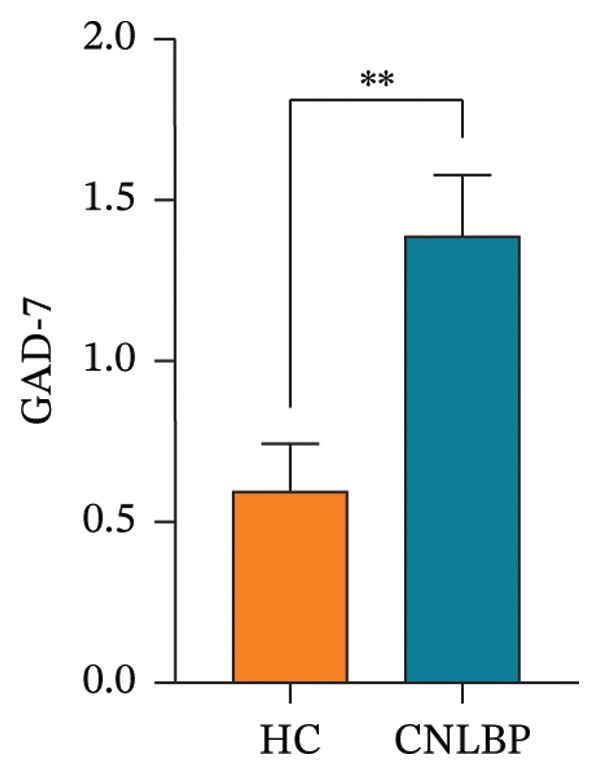
(b)

**Figure:**
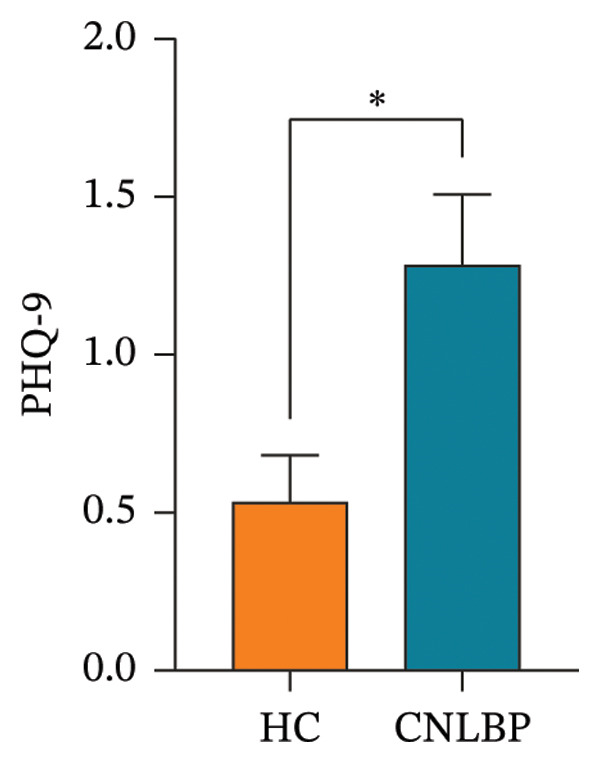
(c)

After PSM, a total of 22 well‐matched sample pairs were obtained. Comparative analysis of the matched data revealed that, except for changes in the significance of FFD (*p* = 0.118) and PSQI (*p* = 0.658), the intergroup differences in neuromuscular indicators directly related to the core pathological mechanisms of the CNLBP group—including the FRR, the CSA of the multifidus, the thickness change rate of the multifidus, and maximum isometric back extension strength—remained entirely consistent with the original analysis in terms of both statistical significance and direction. Complete results of the sensitivity analysis are provided in the supporting information (Supporting Table S1, Figure S1).

In summary, patients with CNLBP exhibit somatic dysfunction characterized by heightened pain sensitivity, decreased muscle function, structural alterations, and abnormal neuromuscular control, accompanied by significant psychological distress and reduced sleep quality.

### 3.3. Correlation Analysis Within the CNLBP Group

Within the CNLBP group, the FRR was negatively correlated with the NRS (*r* = −0.283, *p* = 0.038) and the ODI (*r* = −0.345, *p* = 0.011) (Figures 5(a) and 5(b)), suggesting that reduced muscular coordination and relaxation capacity may simultaneously exacerbate pain perception and limitations in daily activities. In contrast, the multifidus thickness change rate was negatively correlated with the ODI (*r* = −0.285, *p* = 0.037), but its correlation with the NRS was not significant (*r* = −0.172, *p* = 0.214) (Figures 6(a) and 6(b)). The rate of change in multifidus muscle thickness was negatively correlated only with ODI, indicating that impaired muscle contraction function primarily affects physical function directly, whereas pain perception may be more strongly modulated by other factors—such as neural sensitization and psychological expectations—resulting in a nonsignificant correlation with subjective pain sensation. Furthermore, the correlation analysis between musculoskeletal ultrasound indices and sEMG indices revealed that no statistically significant correlation was observed between the CSA of the multifidus muscle and the FRR (*r* = 0.061, *p* = 0.655). However, a significant positive correlation was found between the thickness change rate of multifidus and the FRR (*r* = 0.301, *p* = 0.024) (Figures 7(a) and 7(b)).

FIGURE 5Correlation between the FRR and the ODI and NRS scores in the CNLBP group. (a) Correlation between the FRR and the NRS score; (b) correlation between the FRR and the ODI score. FRR: flexion–relaxation ratio; NRS: numeric rating scale; ODI: Oswestry Disability Index.

**Figure:**
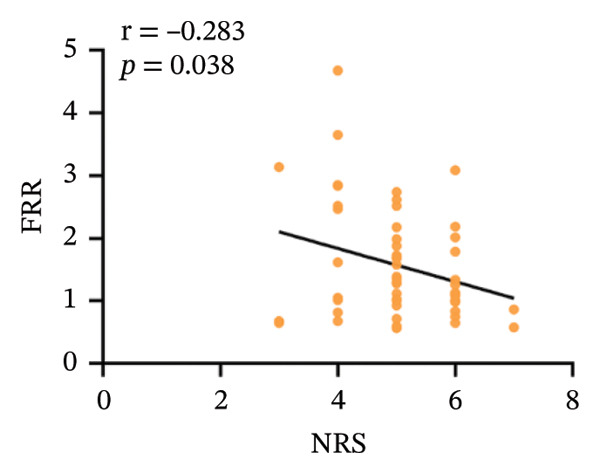
(a)

**Figure:**
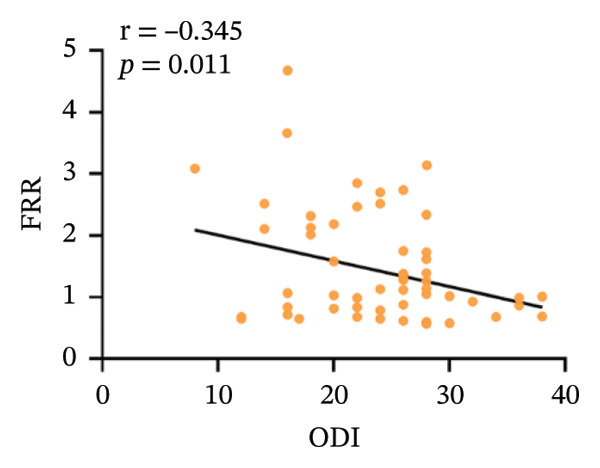
(b)

FIGURE 6Correlation between the rate of change in multifidus muscle thickness and the ODI and NRS scores in the CNLBP group. (a) Correlation between the rate of change in multifidus muscle thickness and the NRS score; (b) correlation between the rate of change in multifidus muscle thickness and the ODI score. NRS: numeric rating scale; ODI: Oswestry Disability Index.

**Figure:**
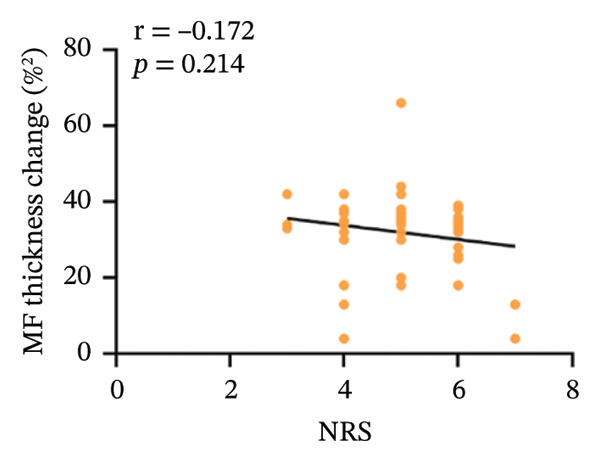
(a)

**Figure:**
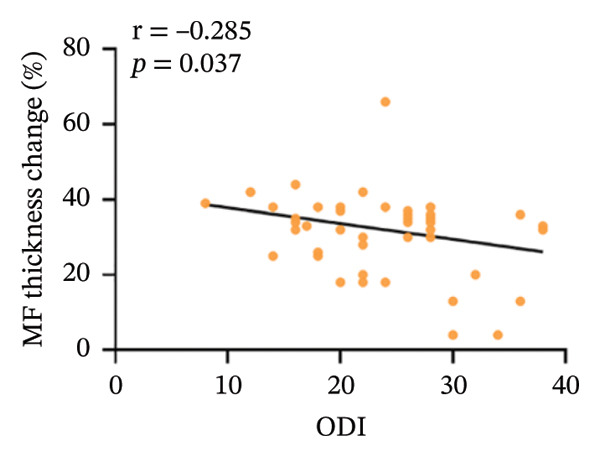
(b)

FIGURE 7Correlation between the MF‐CSA and the rate of change in multifidus muscle thickness and the FRR in the CNLBP group. (a) Correlation between the MF‐CSA and the FRR; (b) correlation between the rate of change in multifidus muscle thickness and the FRR. MF‐CSA: the cross‐sectional area of the multifidus; FRR: flexion–relaxation ratio.

**Figure:**
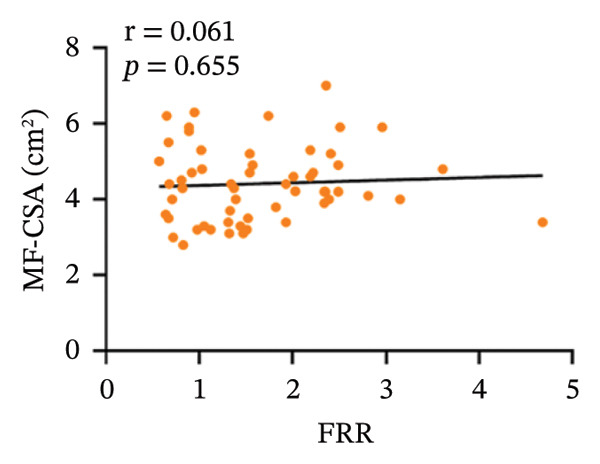
(a)

**Figure:**
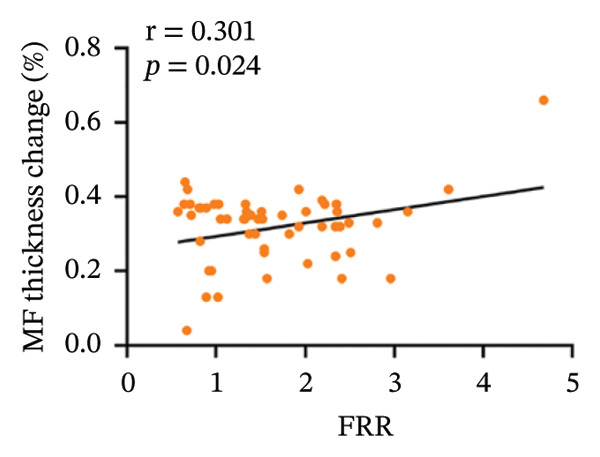
(b)

## 4. Discussion

Through a multidimensional assessment, this study systematically revealed extensive and significant impairments in pain perception and functional performance, core muscle morphology, activation capacity, neuromuscular control, and psychological–emotional status in patients with CNLBP compared to healthy individuals. Further analysis identified significant negative correlations between the FRR and both the NRS and the ODI within the patient cohort. A significant negative correlation was also found between the rate of change in multifidus muscle thickness and the ODI, whereas its correlation with the NRS was not significant. These findings not only deepen the understanding of the complex mechanisms underlying CNLBP from an integrated “symptom–structure–function” perspective but also provide crucial evidence for the clinical development of an objective indicator‐based system for individualized assessment and targeted intervention.

Firstly, this study found that CNLBP patients exhibited decreased back extensor strength, increased FFD, multifidus muscle atrophy, a reduced rate of change in multifidus thickness, and an absence of the flexion–relaxation response in the erector spinae muscles. These changes collectively point toward a failure of the local lumbar stabilization system and a consequent reduction in functional performance. As crucial core stabilizers, structural atrophy, abnormal activation, and impaired flexion–relaxation responses in the multifidus and erector spinae directly compromise the fine control of the spine under both static and dynamic conditions [18, 19]. Research indicates that multifidus atrophy in individuals with CLBP leads to insufficient static and dynamic segmental stability. During dynamic tasks, a reduced rate of change in multifidus thickness represents decreased muscle recruitment efficiency and impaired activation capacity, rendering the spine more susceptible to injury under high loads or in dynamic environments [20, 21]. During lumbar flexion–extension tasks, healthy individuals maintain lumbar stability via segmental stabilizers like the multifidus. At maximum flexion, stress is borne by nonelastic tissues such as bones and ligaments [22], while the superficial erector spinae enter an “electrical silence” or resting state—an efficient energy‐saving mechanism allowing muscle rest [23]. In contrast, the CNLBP group in this study demonstrated a reduced FRR, manifesting as a diminished or absent flexion–relaxation phenomenon. To maintain spinal homeostasis, the erector spinae must initiate compensatory contractions. Prolonged compensation can trigger fiber‐type transformation in the erector spinae, ultimately leading to reduced overall back extensor strength [24]. This may increase abnormal segmental stress during daily activities, serving both as a source of pain and potentially exacerbating multifidus muscle inhibition and atrophy. The reduction in the rate of multifidus thickness change, used as an indicator of muscle activation level, indicates that multifidus contraction is suppressed [21]. Previous studies have also indicated that in the pain adaptation model, muscle contraction is often inhibited to reduce pain perception, leading to compensatory actions by other muscle subsystems to enhance the overall stability of the spinal system. This phenomenon is often interpreted as a protective muscle coactivation compensation or an altered neuromotor control strategy by the spinal musculoskeletal system under the threat of pain [25], creating a “splinting” effect that limits lumbar mobility in CNLBP patients, thereby contributing to increased FFD. Extensive research has confirmed the presence of multifidus atrophy in individuals with chronic LBP , a condition that can further contribute to spinal instability and potential injury [26, 27].

Secondly, our analysis also found that compared to healthy individuals, CNLBP patients had lower PPT and commonly presented with significant anxiety, depressive moods, and reduced sleep quality, suggesting CNLBP as a psychosomatic interactive disorder. Negative emotions and sleep deprivation can lower pain thresholds and increase muscle tension via neuroendocrine mechanisms, while persistent pain and dysfunction, in turn, exacerbate psychological distress [28–30]. Recent studies show that compared to patients with axial spondyloarthritis, CNLBP patients experience more severe pain, more significant functional disability, and poorer quality of life, highlighting the substantial disease burden imposed by CNLBP as a distinct clinical entity [31]. This multisystem interaction collectively forms the basis for the chronicity and refractoriness of CNLBP.

Thirdly, the results of this study showed significant negative correlations between FRR and both NRS scores and ODI. This finding underscores the central role of neuromuscular control function in the pathological progression of CNLBP. According to Panjabi’s three‐dimensional theory of spinal stability, maintaining spinal homeostasis relies not only on active components like muscles and passive components like ligaments and intervertebral discs but also crucially on the sensorimotor integration function of the neural control unit as the core regulatory link [32]. Correlation analysis revealed that the degree of neuromuscular control impairment, represented by FRR, is significantly associated with clinical experiences such as pain perception and limitations in daily activities. A previous study also confirmed a high correlation between FRR and disability caused by CLBP [33]. This positions FRR as a potential quantifiable biomarker for assessing the pathological progression of neuromuscular control in CNLBP, providing an objective basis for moving beyond traditional single‐dimensional pain assessment models [34]. It also suggests a new direction for clinical intervention in CNLBP: Rehabilitation training should not only focus on strengthening muscle power, dimensions, and joint range of motion but also prioritize the restoration of efficient and coordinated neuromuscular control patterns. Randomized controlled trials have already confirmed that targeted interventions, such as proprioceptive neuromuscular facilitation and dynamic neuromuscular stabilization training, can significantly improve FRR levels in CNLBP patients while alleviating pain and dysfunction [35, 36], which aligns closely with the intervention direction proposed in this study. The core mechanism lies in how such training can remodel sensorimotor integration pathways, repair the central nervous system’s fine‐tuning control over spinal muscles, and break the vicious cycle of “pain–muscle control impairment–worsening pain.”

Fourthly, the study found that the rate of change in multifidus thickness was significantly correlated only with ODI scores and not with NRS pain intensity. This result clearly distinguishes the regulatory pathways governing dynamic muscle function and pain perception. As a deep core spinal muscle, the dynamic contraction capacity of the multifidus directly determines the dynamic stability of lumbar segments. It maintains spinal biomechanical balance by fine‐tuning intervertebral motion and distributing loads across vertebral bodies. Therefore, its functional impairment primarily affects the execution efficiency of functional tasks like bending and twisting [26]. In contrast, a patient’s immediate pain perception is more readily modulated by factors such as emotional state, pain expectancy, and the degree of central sensitization [37]. Under central sensitization, increased excitability of dorsal horn neurons and abnormal activation of glial cells releasing inflammatory factors can significantly amplify pain signal transmission, leading to exaggerated pain responses to minor stimuli [38]. This mechanism has been confirmed by functional magnetic resonance imaging studies, which show that abnormal neural activity in pain matrix‐related brain regions (e.g., the precuneus) in CNLBP patients is directly related to the degree of pain sensitization [39]. In clinical studies involving subjective human indicators such as pain and functional scales, a single objective measurement typically explains only a portion of the symptom variability. The coefficient of determination (*R* ^2^) corresponding to our r‐values ranges from approximately 0.08 to 0.12, indicating that it can account for about 8%–12% of the variation in pain or functional impairment. This aligns with the current understanding of the multidimensional and complex etiology of NSLBP, where pain and dysfunction are also influenced by a combination of biological, psychological, and social factors [40]. Therefore, a moderate rather than a strong correlation accurately reflects the nature of CNLBP as a complex syndrome.

Finally, upon further analysis, this study found no significant correlation between the CSA of the multifidus muscle and the FRR, whereas a significant positive correlation was observed between the rate of change in multifidus thickness and the FRR. This indicates that mere morphological muscle atrophy does not directly equate to the degree of abnormality in the nervous system’s control of movement, and vice versa. It also supports the possibility that the core dysfunction in CNLBP may stem more from the interaction between impaired muscle activation efficiency and abnormal motor control patterns, rather than from static muscle atrophy itself. This finding may hold certain implications for clinical practice. First, ultrasound, as a safe and noninvasive diagnostic tool, has been proven to be a reliable method for evaluating skeletal muscle structure [41]. sEMG can quantitatively assess neuromuscular control function. The combination of these two methods allows for a multidimensional understanding of the pathological mechanisms of CNLBP from static and dynamic, structural, functional, and control perspectives. This integrated approach is also uniquely significant for clarifying the specific pathological changes in each CNLBP patient, thereby facilitating targeted interventions. Therefore, the incorporation of both assessment types into the evaluation system should be considered. Second, while numerous classification systems for LBP currently exist, issues such as overlapping or unclear categories are inevitable. This study offers a new perspective for the classification and subtyping of LBP. Third, during the rehabilitation process, these metrics can separately quantify improvements in the static structure and dynamic function of the local multifidus muscle, as well as the level of neural control of the back erector spinae. This enables more comprehensive, objective, and timely feedback on therapeutic efficacy compared to relying solely on a single pain scale or a functional questionnaire. This also suggests to clinicians that, while improvements in neuromuscular control may have a certain degree of positive impact on pain relief and functional enhancement, these should be integrated with interventions targeting other pathogenic factors to achieve optimal therapeutic outcomes.

Distinguishing between these functional and pain regulatory pathways holds significant clinical practical importance. It clarifies that “improving function” and “alleviating pain” require sequential intervention strategies with different emphases. For functional disability caused by insufficient dynamic contraction capacity of the multifidus, active rehabilitation programs centered on precise multifidus activation training—such as targeted contraction training guided by ultrasound—should be prioritized to enhance dynamic muscle contraction efficiency. For neuromuscular control dysfunction, integrated training approaches like proprioceptive neuromuscular facilitation should be prioritized to address the pain state, among other factors.

This analysis included several limitations: Firstly, regarding the imbalance in sample size and baseline characteristics (such as smoking status), while this deviates from ideal conditions, it stems from the practical recruitment context. The robustness of the main conclusions has been strengthened through the appropriate statistical methods and additional sensitivity analyses (e.g., PSM) employed during the analysis phase. Secondly, its cross‐sectional design precludes causal inferences. Future high‐quality longitudinal studies are needed to elucidate the temporal relationship between these abnormal indicators and the onset and progression of CNLBP. Finally, due to the limited sample size and the small number of individuals in certain subgroups (e.g., HCs who are current smokers, *n* = 3), the feasibility of conducting in‐depth subgroup analyses on factors such as smoking status was significantly constrained in this study. Therefore, it is necessary to validate the influence of these potential factors in larger samples in the future.

## 5. Conclusion

The results of this study confirmed that in addition to pain and functional impairment, patients with CNLBP also exhibit changes such as multifidus muscle atrophy, a decreased rate of change in multifidus muscle thickness, and a reduced FRR of the erector spinae muscles. Among these, the rate of change in multifidus muscle thickness was negatively correlated only with the ODI, while the FRR was negatively correlated with both the NRS and the ODI. Future research should focus on developing targeted treatment plans centered on restoring neuromuscular control and validating their long‐term efficacy through well‐designed randomized controlled trials, with the aim of genuinely improving the long‐term prognosis of patients with CNLBP.

## Author Contributions

Zhao Wang drafted the manuscript and conducted statistical analysis. Fen Ju was responsible for collecting the questionnaire data and writing the methods section. Dexin Hu contributed to the background writing. Yuheng Lu was responsible for data collection. Chenguang Zhao was responsible for guiding and reviewing this article.

## Funding

This work was supported by the Shaanxi Science and Technology Foundation (grant no. 2024JC‐YBMS‐620).

## Disclosure

All authors have read and approved the final manuscript.

## Ethics Statement

This study was approved by the Ethics Committee of Xijing Hospital, the Fourth Military Medical University (Approval No.: KY20242185‐F‐1) and all participants provided written informed consent.

## Conflicts of Interest

The authors declare no conflicts of interest.

## Supporting Information

Additional supporting information can be found online in the Supporting Information section.

## Supporting information


**Supporting Information** The following supporting file is available online (Supporting Materials). Figure S1: Standardized difference plot. Table S1: Comparison of related variables between the HC and CNLBP groups after propensity score matching.

## Data Availability

The datasets generated and analyzed during the current study are not publicly available due to ethical restrictions and the protection of participant privacy. The data contain sensitive personal health information. However, deidentified data can be made available from the corresponding author upon reasonable request and with the approval of the institutional ethics committee.
