# Supplementary material for: Multidimensional Dysfunction in Chronic Nonspecific Low Back Pain: A Correlational Study of Key Clinical Measures
Source: Pain Res Manag. 2026 May 30;2026:4984566. doi: 10.1155/prm/4984566 (PMC13239188; doi:10.1155/prm/4984566)
Supplement: Supplementary file 1 — Supporting Information The following supporting file is available online (Supporting Materials). Figure S1: Standardized difference plot. Table S1: Comparison of related variables between the HC and CNLBP groups after propensity score matching. [file PRM-2026-4984566-s001.docx]

| **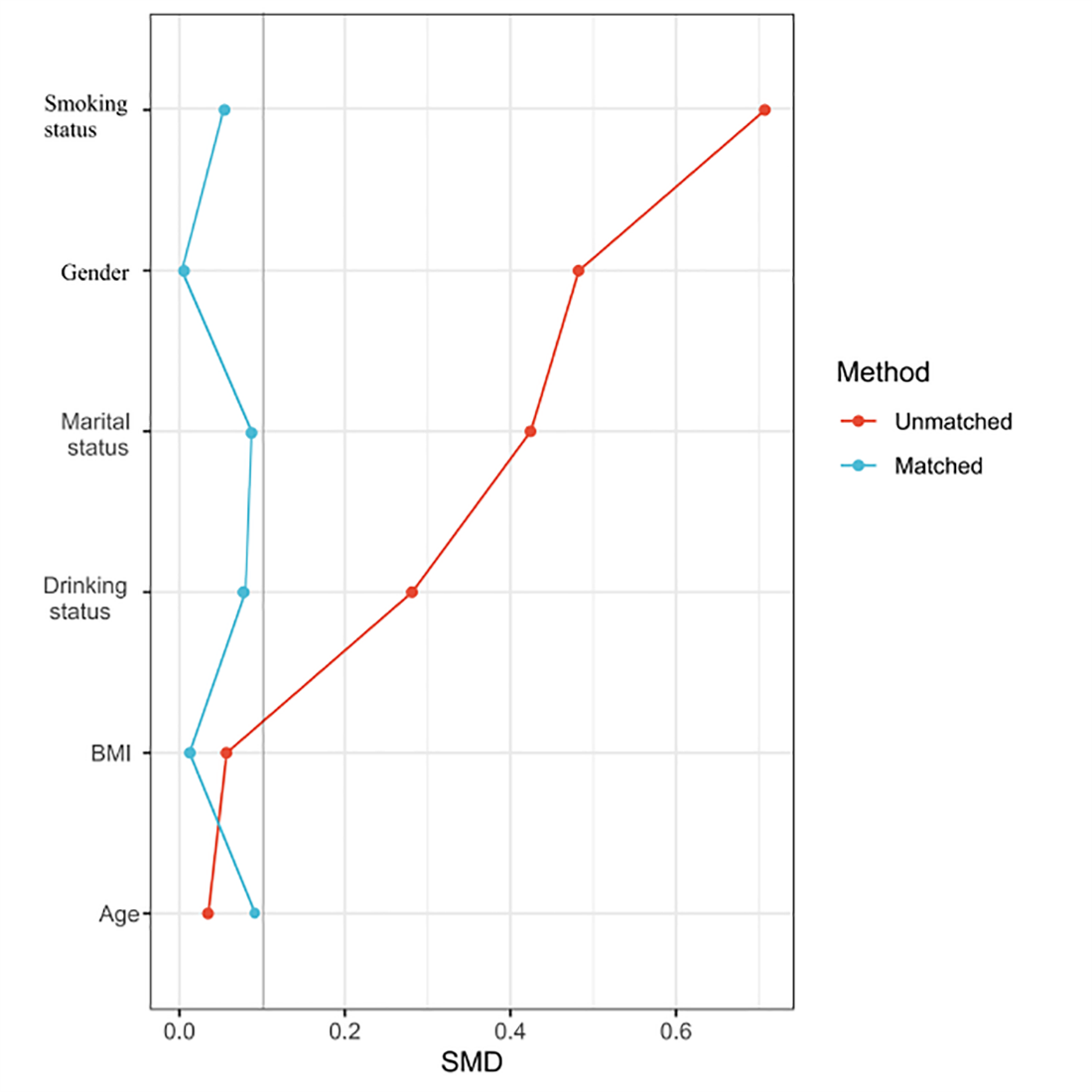** |
| --- |
| **Figure S1.** Standardized Difference Plot. |

**Table S1. Comparison of related variables between the HC and CNLBP groups after propensity score matching**

| Items | HC group  N = 22 (50%) | CNLBP group  N = 22 (50%) | *p*-value |
| --- | --- | --- | --- |
| PPT (kg), Mean ± SD | 2.62±0.91 | 2.07±0.93 | 0.017 |
| MIBES (N), Mean ± SD | 206.23±14.66 | 182.64±17.06 | <0.001 |
| FFD (cm), Mean ± SD | 12.84±5.46 | 15.64±6.13 | 0.118 |
| MF-CSA (cm^2^), Mean ± SD | 6.01±1.73 | 4.43±0.90 | <0.001 |
| MF thickness change rate (%), Mean ± SD | 42.00±10.00 | 32.00±9.00 | 0.002 |
| FRR, Mean ± SD | 4.56±1.65 | 1.54±0.71 | <0.001 |
| PSQI, Mean ± SD | 2.88±1.82 | 3.07±1.67 | 0.658 |
| GAD-7, Mean ± SD | 0.68±0.62 | 1.75±1.33 | 0.005 |
| PHQ-9, Mean ± SD | 0.52±0.75 | 1.32±1.8 | 0.048 |

Note: Comparisons were made using independent samples t-test. HC: healthy control; CNLBP: chronic non-specific low back pain; PPT: pressure pain threshold; MIBES: maximal isometric back extensor strength; FFD: finger to floor distance; MF-CSA: the cross-sectional area of the multifidus; FRR: flexion-relaxation ratio; PSQI: Pittsburgh sleep quality index; GAD-7: generalized anxiety disorder 7; PHQ-9: patient health questionnaire-9.
